# Outcomes in growth hormone-treated Noonan syndrome children: impact of *PTPN11* mutation status

**DOI:** 10.1530/EC-21-0615

**Published:** 2022-03-04

**Authors:** Alexander A L Jorge, Thomas Edouard, Mohamad Maghnie, Alberto Pietropoli, Nicky Kelepouris, Alicia Romano, Martin Zenker, Reiko Horikawa

**Affiliations:** 1 Unidade de Endocrinologia-Genetica, LIM/25, Disciplina de Endocrinologia da Faculdade de Medicina da Universidade de Sao Paulo (FMUSP), Sao Paulo, Brazil; 2 Endocrine, Bone Diseases, and Genetics Unit, Children’s Hospital, Toulouse University Hospital, RESTORE INSERM UMR1301, Toulouse, France; 3 Department of Pediatrics, IRCCS Istituto Giannina Gaslini, Genova, Italy; 4 Department of Neuroscience, Rehabilitation, Ophthalmology, Genetics, Maternal and Child Health, University of Genova, Genova, Italy; 5 Novo Nordisk Health Care AG, Global Medical Affairs Biopharm, Zürich, Switzerland; 6 Novo Nordisk Inc., Clinical, Medical and Regulatory Biopharm-RED, Plainsboro, New Jersey, USA; 7 Department of Pediatrics, New York Medical College, Valhalla, New York, USA; 8 Institute of Human Genetics & Department of Pediatrics, University Hospital, Otto-von-Guericke University Magdeburg, Magdeburg, Germany; 9 Department of Endocrine and Metabolism, National Center for Child Health and Development, Tokyo, Japan

**Keywords:** growth hormone, Noonan syndrome, PTPN11, RASopathies, short stature

## Abstract

**Introduction:**

Mutations in *PTPN11* are associated with Noonan syndrome (NS). Although the effectiveness of growth hormone therapy (GHT) in treating short stature due to NS has been previously demonstrated, the effect of *PTPN11* mutation status on the long-term outcomes of GHT remains to be elucidated.

**Methods:**

This analysis included pooled data from the observational American Norditropin Studies: Web-Enabled Research Program (NCT01009905) and the randomized, double-blinded GHLIQUID-4020 clinical trial (NCT01927861). Pediatric patients with clinically diagnosed NS and confirmed *PTPN11*mutation status were eligible for inclusion. The effectiveness analysis included patients who were GHT-naïve and pre-pubertal at GHT start. Growth outcomes and safety were assessed over 4 years of GHT (Norditropin®, Novo Nordisk A/S).

**Results:**

A total of 69 patients were included in the effectiveness analysis (71% *PTPN11* positive). The proportion of females was 32.7 and 30.0% in *PTPN11*-positive and negative patients, respectively, and mean age at GHT start was 6.4 years in both groups. Using general population reference data, after 4 years of GHT, the mean (s.d.) height SD score (HSDS) was −1.9 (1.1) and −1.7 (0.8) for *PTPN11*-positive and *PTPN11*-negative patients, respectively, with no statistical difference observed between groups. The mean (s.d.) change in HSDS at 4 years was +1.3 (0.8) in *PTPN11*-positive patients and +1.5 (0.7) in *PTPN11-*negative patients (no significant differences between groups). Safety findings were consistent with previous analyses.

**Conclusions:**

GHT resulted in improved growth outcomes over 4 years in GHT-naïve, pre-pubertal NS patients, irrespective of *PTPN11* mutation status.

## Introduction

Noonan syndrome (NS) is a genetically heterogenous disorder characterized by various clinical features including cardiovascular abnormalities, short stature, distinctive facial features, skeletal anomalies, cryptorchidism in boys, bleeding, ophthalmologic and hearing issues, mild developmental delay/intellectual disability, and a predisposition to malignancy (1, 2, 3, 4). The presentation of symptoms varies among individuals, and the individual health impairment can range from mild to severe. It is estimated that the prevalence of NS is approximately 1 in 1000 to 1 in 2500 live births (4).

NS is usually inherited in an autosomal dominant pattern, but autosomal recessive inheritance also occurs as a result of biallelic mutations in the leucine zipper-like transcription regulator 1 (*LZTR1*) gene (2, 4, 5). However, in the majority of cases, the disease-causing dominant mutation arises *de novo* (2, 4). Several genes encoding proteins of the RAS/mitogen-activated protein kinase (MAPK) signaling pathway have also been identified as NS causative genes, most commonly due to gain-of-function mutations. Mutations most commonly occur in the *PTPN11* gene, accounting for approximately 50–60% of mutations identified in NS patients (2, 6). However, in around 20–30% of cases, the underlying mutation has not yet been identified (6, 7), although this proportion can depend upon the stringency of the criteria for clinical diagnosis (2).

Although children with NS typically have a birth weight and length within the normal range, patients often suffer postnatal growth failure within the first years of life, and approximately 60–70% of children with NS are subsequently affected by short stature (1, 8, 9, 10). In addition, puberty is often delayed and is accompanied by a delay in bone age (1, 9, 11). It is estimated that over 50% of females and almost 40% of males with NS have an adult height below the third percentile (1).

Abnormalities of growth hormone (GH) secretory dynamics in NS are inconsistent and may include growth neurosecretory dysfunction, GH deficiency (GHD), and GH insensitivity (12, 13, 14, 15, 16). Increased activation of the RAS/MAPK pathway has been demonstrated to cause a reduction in growth plate length in NS mice (17). A higher prevalence of short stature has been reported among NS patients with a mutation in *PTPN11*or *RAF1*, while a lower prevalence of short stature has been reported among patients with a *SOS1* mutation (9, 18).

Norditropin® (somatropin; Novo Nordisk A/S) is a recombinant human GH (rhGH) that is used as replacement therapy in patients with GHD or to treat a number of other conditions characterized by insufficient growth (US Food and Drug Administration - Norditropin, highlights of prescribing information, https://www.accessdata.fda.gov/drugsatfda_docs/label/2018/021148s037s038lbl.pdf; Norditropin® summary of product characteristics, https://www.medicines.org.uk/EMC/medicine/2760/SPC/Norditropin+SimpleXx+5+mg+1.5+mL%2c+10+mg+1.5+mL%2c+15+mg+1.5+mL%3b+Norditropin+NordiFlex+15+mg+1.5+mL). Norditropin® is currently the only GH replacement therapy approved internationally for the treatment of short stature in children with NS (in the United States, European Union, Japan, Israel, Brazil, South Korea, Switzerland, Canada, and Argentina). A small number of studies have investigated the effect of *PTPN11* mutation status on response to GH therapy (GHT), but findings to date have been discordant (8, 14, 19, 20). This analysis aimed to further evaluate the impact of *PTPN11* mutation status on long-term effectiveness and safety outcomes in pre-pubertal NS patients receiving GHT.

## Materials and methods

### Data source

**Figure 1:**
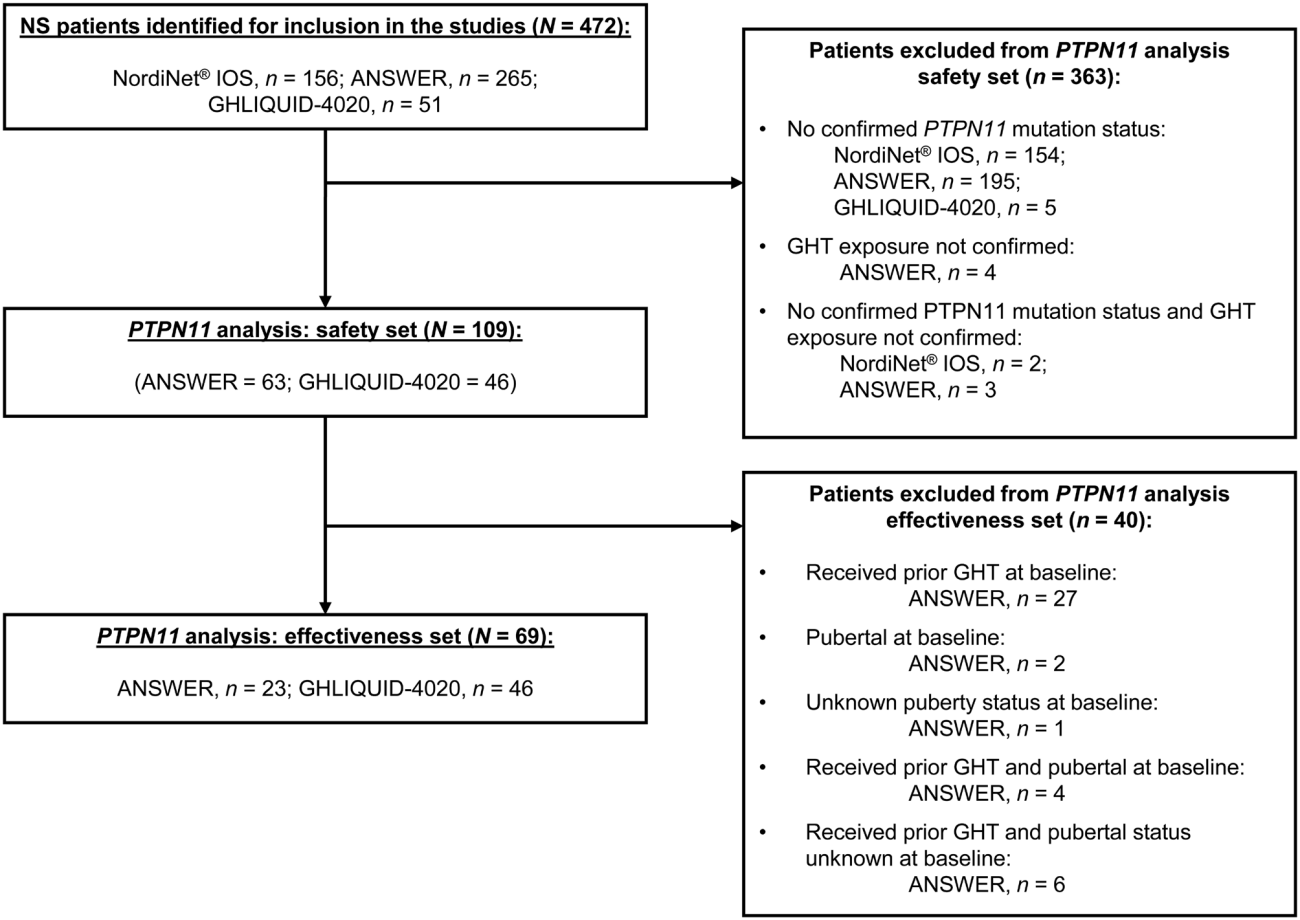
Patient disposition. ANSWER, American Norditropin Studies: Web-Enabled Research; GHT, growth hormone therapy; IOS, International Outcome Study; NS, Noonan syndrome.

This was an analysis of a subset of pooled data from the observational American Norditropin Studies: Web-Enabled Research (ANSWER) Program® (NCT01009905) and the GHLIQUID-4020 clinical trial (NCT01927861). The design of these studies has been previously described in detail (21, 22, 23). The current analysis used data from a subset of NS patients enrolled in the ANSWER program® and GHLIQUID-4020, including only patients with confirmed *PTPN11* mutation status. Data from the Nordinet® International Outcome Study (IOS) were not included in this analysis as *PTPN11* mutation status was not confirmed in NS patients enrolled in this study (shown in Fig. 1).

In brief, the ANSWER program® was an observational, multicenter study conducted across 207 USA centers between 2002 and 2016, which monitored the long-term effectiveness and safety of rhGH therapy (Norditropin® (somatropin); Novo Nordisk A/S) in routine clinical practice (23). Adult and pediatric patients with a variety of conditions affecting growth were eligible for inclusion, including patients with NS. All visits were carried out according to routine clinical practice and doses of rhGH were prescribed by the treating physician according to standard clinical practice. Effectiveness endpoints for pediatric patients included height gain, body weight, insulin-like growth factor I (IGF-I) level, and bone age. Adverse drug reactions (ADR), serious ADRs (SADR), and serious adverse events (SAE) were recorded for all patients throughout the duration of treatment.

The GHLIQUID-4020 trial was a 4-year multicenter, randomized, parallel group, double-blinded trial investigating the long-term efficacy and safety of rhGH treatment (Norditropin®) in short-statured Japanese children with NS (23). The study was conducted in 26 Japanese centers between 2013 and 2018. Pre-pubertal NS children, with a height standard deviation score (HSDS) ≤−2 according to Japanese national reference data (24), were eligible for inclusion. Eligible children were aged 3–<11 years for boys and 3–<10 years for girls. Patients were randomized 1:1 to receive a daily rhGH dose of 0.033 or 0.066 mg/kg. The primary endpoint was change in HSDS from baseline to 104 weeks of treatment. Patients were given the option of continuing treatment for a further 104 weeks in an extension to the trial (208 weeks of treatment in total). Safety measurements were recorded for all patients at each visit for the duration of the trial.

Approval for both studies was obtained from relevant ethics committees and all patients (and/or their guardians where the patient was a minor) provided written informed consent. The studies were conducted in accordance with the Declaration of Helsinki and all data included in the analysis were anonymized.

### Study population

The current analysis included pooled data from pediatric patients with clinically diagnosed NS and confirmed *PTPN11*mutation status, who were enrolled in the above studies. Patients were categorized into 1 of 2 groups on the basis of genetic test results: *PTPN11* positive or *PTPN11* negative. The *PTPN11*-negative group included patients with other confirmed genetic etiologies and unsolved cases, in addition to a negative *PTPN11* test.

### Study outcomes

The descriptive analysis included the following parameters: sex, other identified genetic mutations (for patients without *PTPN11* mutation), age at GHT start, rhGH dose, HSDS, BMI SD score (SDS), bone age, bone age/chronological age ratio, IGF-I SDS, and cardiovascular comorbidities. The primary effectiveness analysis was long-term growth outcome over 4 years of GHT, as measured by HSDS, and change in HSDS from GHT start (baseline). Two HSDS values were calculated; the first using general population reference growth charts and the second using NS-specific growth charts as the reference. For the ANSWER program®, Centers for Disease Control and Prevention (CDC) national reference growth charts were used for the general population HSDS (25) and growth charts as reported by Ranke *et al.* were used to calculate NS-specific HSDS (26). For the GHLIQUID-4020 trial, Japanese national reference data for children (24) were used to calculate the general population HSDS and data as reported by Isojima *et al.* were used to calculate NS-specific HSDS (27). BMI SDS was derived from height and weight measurements and country-specific reference tables, as reported by Inokuchi *et al.* for the GHLIQUID-4020 trial (28) and using CDC general population reference growth charts for the ANSWER program® (25). Safety was also assessed; non-serious and SADRs and SAEs were reported for the overall population. Adverse events that were considered possibly or probably related to rhGH treatment, either by the reporting physician or the study sponsor, were considered ADRs.

### Statistical analysis

Patient characteristics and baseline variables were summarized using mean and s.d. for continuous variables and relative frequencies and proportions for categorical variables. Patients included in the safety analysis set had clinically diagnosed NS and confirmed* PTPN11* mutation status. The effectiveness analysis set was a subset of the safety analysis set, including only patients who were rhGH treatment-naïve and pre-pubertal upon entry to the studies (baseline). Data were analyzed using SAS software version 9.4, and significance testing was carried out using a *t*-test or Mann–Whitney *U* test, dependent upon whether the normality assumption was met.

## Results

### Patient disposition

Patient disposition is outlined in Fig. 1. In total, 472 pediatric patients with clinically diagnosed NS were identified from the studies. Of these, 109 patients had confirmed rhGH exposure and documented *PTPN11* mutation status and were included in the safety analysis set. The effectiveness analysis set included 69 patients with confirmed *PTPN11* mutation status and who were also rhGH treatment-naïve and pre-pubertal at baseline. The most common reason for exclusion from the safety analysis set was the absence of genetic testing; no patients from the Nordinet® IOS were included in the analysis for this reason.

### Baseline characteristics

Patient characteristics at baseline are shown in Table 1. In total, 49 patients (71%) were *PTPN11* positive and 20 patients (29%) were *PTPN11* negative. Baseline characteristics were generally similar between *PTPN11*-positive and *PTPN11*-negative patients, with no significant differences identified in any of the variables. The mean (s.d.) age at GHT start was 6.4 (3.3) years in *PTPN11-*positive patients and 6.4 (2.5) years in *PTPN11*-negative patients, and the proportion of females in each group was 32.7 and 30.0%, respectively. Provocative GH testing results were unavailable for all 69 patients; tests were either not performed or results not reported. Mean (s.d.) rhGH dose at baseline was 0.047 (0.015) mg/kg/day in* PTPN11-*positive patients and 0.054 (0.016) mg/kg/day in *PTPN11*-negative patients. In total, 42.9% of *PTPN11*-positive patients and 50.0% of* PTPN11*-negative patients reached puberty during the 4-year study period.

**Table 1:** Patient characteristics at baseline by *PTPN11* mutation status (effectiveness population, *n*  = 69).

	*PTPN11*+ (*n*= 49)		*PTPN11*– (*n* = 20)	
	*n*		*n*	
Female, *n* (%)	49	16 (32.7)	20	6 (30.0)
Age at GH start, years, mean (s.d.)	49	6.4 (3.3)	20	6.4 (2.5)
GH dose at baseline, mg/kg/day, mean (s.d.)	48	0.047 (0.015)	20	0.054 (0.016)
Height SDS (general population), mean (s.d.)	49	−3.0 (0.8)	20	−3.1 (0.8)
Height SDS (NS specific), mean (s.d.)	48	−0.5 (0.8)	20	−0.6 (0.8)
BMI SDS, mean (s.d.)	47	−0.6 (1.3)	20	0.0 (1.1)
Bone age, mean (s.d.)	39	5.6 (2.6)	20	5.4 (2.7)
Bone age/chronological age, mean (s.d.)	39	0.9 (0.3)	20	0.8 (0.2)
IGF-I SDS, mean (s.d.)	35	−1.5 (1.3)	20	−1.2 (0.8)

*n,* number of patients with available data.

GH, growth hormone; IGF-I, insulin-like growth factor I; NS, Noonan syndrome; SDS, standard deviation score.

Of the 20 patients who were *PTPN11* negative, 9 had an NS-causative mutation identified in a different gene, while for 11 patients, no causative mutation was reported. Mutations identified included* RAF1* (*n*  = 3), *SOS1* (*n*  = 2), *BRAF*(*n*  = 1), *KRAS*(*n*  = 1), *RIT1*(*n*  = 1), and *SHOC2*(*n*  = 1).

In total, cardiovascular comorbidities were present in 55.1% of patients at baseline. The number of cardiovascular comorbidities recorded was similar between *PTPN11*-positive and *PTPN11*-negative patients. Of the patients with reported cardiovascular comorbidity, most patients had one (20.4% of *PTPN11*-positive patients vs 20.0% of *PTPN11*-negative patients) or two cardiovascular comorbidities reported (24.5% vs 25.0%, respectively). The most commonly reported cardiovascular comorbidities were atrial septal defect (recorded in 23.2% of all patients), pulmonary valve stenosis (17.4%), pulmonary artery stenosis (10.1%), and hypertrophic cardiomyopathy (10.1%). Cardiovascular phenotypes were similar between *PTPN11*-positive and *PTPN11*-negative patients at baseline.

### Growth outcomes

**Figure 2:**
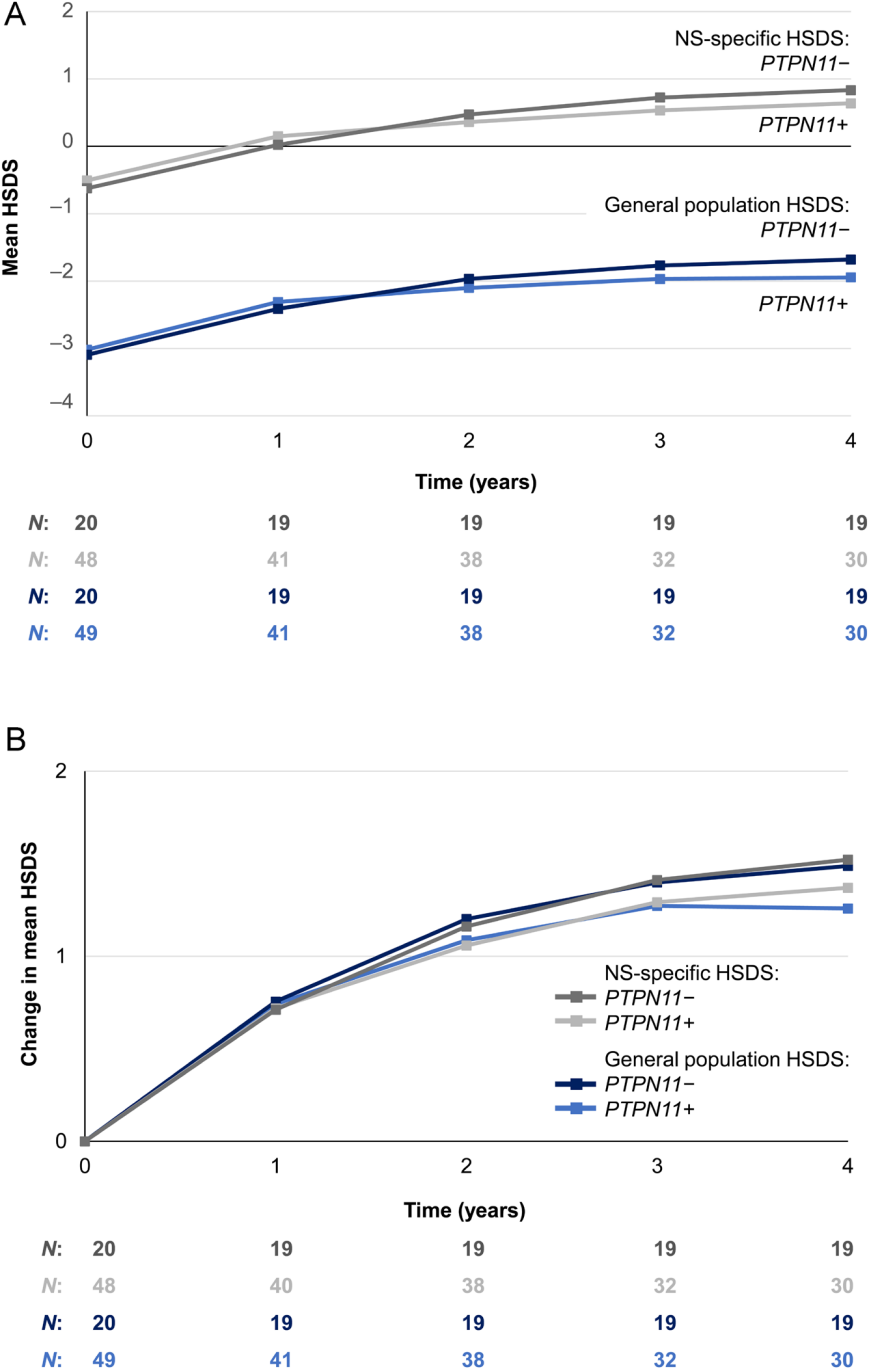
(A) HSDS by year from baseline and (B) improvement in HSDS by year from baseline (effectiveness population, *n*  = 69). *n*, number of patients with available data at each time point. HSDS (general population reference) was calculated using CDC national reference growth charts (27) for the ANSWER program® and using Japanese national reference data for the GHLIQUID-4020 trial (26). HSDS (NS-specific) was calculated using reference data reported by Ranke *et al.* (28) for the ANSWER program® and using data reported by Isojima *et al.* (29) for the GHLIQUID-4020 trial. ANSWER, American Norditropin Studies: Web-Enabled Research; CDC, Centers for Disease Control and Prevention; NS, Noonan syndrome; (H) SDS, (height) standard deviation score.

HSDS by year from GHT start and change in HSDS from GHT start are shown in Fig. 2A and B, respectively. Using general population reference data, mean (s.d.) HSDS at baseline was −3.0 (0.8) among *PTPN11*-positive patients and −3.1 (0.8) among *PTPN11-*negative patients. After 4 years of rhGH treatment, mean (s.d.) HSDS was −1.9 (1.1) and −1.7 (0.8) for *PTPN11*-positive and *PTPN11*-negative patients, respectively, with no statistical difference between groups (*P* = 0.3518). Using NS-specific reference data, mean (s.d.) HSDS at baseline was −0.5 (0.8) among *PTPN11*-positive patients and −0.6 (0.8) among *PTPN11-*negative patients. Following 4 years of treatment, mean (s.d.) HSDS was 0.6 (1.0) and 0.8 (0.8) among *PTPN11*-positive and *PTPN11-*negative patients, respectively, with no statistical difference between groups (*P* = 0.4551).

The mean (s.d.) change in HSDS at 4 years from baseline was +1.3 (0.8) for *PTPN11*-positive patients and +1.5 (0.7) for *PTPN11-*negative patients (based on general population reference data). Based on NS-specific reference data, the mean (s.d.) change in HSDS at 4 years from baseline was similar (+1.4 (0.8) and +1.5 (0.7) for *PTPN11*-positive and *PTPN11-*negative patients, respectively). No significant differences in HSDS change from baseline were observed between *PTPN11*-positive and *PTPN11-*negative patients (for both HSDS methods).

**Figure 3:**
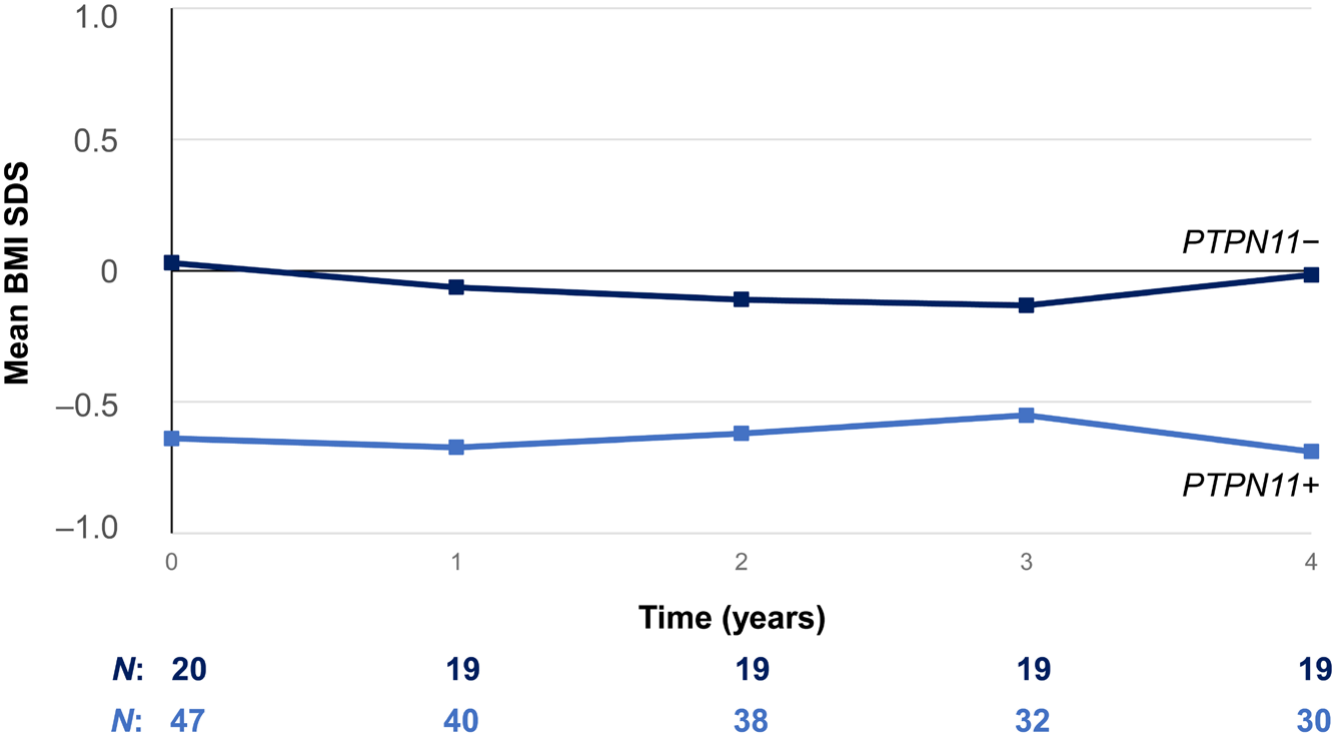
Change in BMI SDS from baseline (effectiveness population, *n*  = 69). *n*, number of patients with available data at each timepoint. BMI SDS was calculated using country-specific reference tables, as reported by Inokuchi *et al.* for the GHLIQUID-4020 trial (30) and CDC general population reference growth charts for the ANSWER program® (27). ANSWER, American Norditropin Studies: Web-Enabled Research; CDC, Centers for Disease Control and Prevention; SDS, standard deviation score.

The change in BMI SDS from baseline for *PTPN11-*positive and negative patients is shown in Fig. 3. Mean (s.d.) BMI SDS at baseline was −0.64 (1.26) for *PTPN11-*positive patients and 0.03 (1.08) for *PTPN11-*negative patients (*P* = 0.0421). After 4 years of GHT, mean (s.d.) BMI SDS was −0.69 (1.07) and −0.02 (0.94) for the *PTPN11-*positive and *PTPN11-*negative patients, respectively (*P* = 0.0294). There were no significant differences between *PTPN11-*positive and *PTPN11*-negative patients in the change in BMI SDS from baseline (−0.02 vs −0.04, respectively).

### IGF-I

IGF-I SDS by *PTPN11* mutation status and rhGH dose for patients included in the GHLIQUID-4020 trial (*n*  = 46) is shown in Table 2. Irrespective of *PTPN11* mutation status, IGF-I levels did not exceed the normal range (+2 SDs) for either rhGH dose. *PTPN11-*positive patients receiving a dose of 0.066 mg/kg/day had IGF-I SDS levels in the positive range, with a mean (s.d.) change from baseline at 4 years of +2.3 (1.0). Among *PTPN11*-positive patients receiving a dose of 0.033 mg/kg/day, IGF-I SDS levels remained below 0, with a mean (s.d.) change from baseline at 4 years of +0.8 (0.8). The difference between IGF-I SDS levels at 4 years between *PTPN11*-positive and *PTPN11*-negative patients (0.033 mg/kg daily dose) were significant (*P*  = 0.0037), but there were no significant differences in the change from baseline in IGF-I SDS between *PTPN11-*positive and negative patients.

**Table 2:** IGF-I SDS by GH dose and *PTPN11* mutation status for patients (GHLIQUID-4020 trial effectiveness population, *n*  = 46).

	Year	rhGH dose: 0.033 mg/kg/day									rhGH dose: 0.066 mg/kg/day								
		*PTPN11*+			*PTPN11*–			All			*PTPN11*+			*PTPN11*–			All		
		*n*	Mean	s.d.	*n*	Mean	s.d.	*n*	Mean	s.d.	*n*	Mean	s.d.	*n*	Mean	s.d.	*n*	Mean	s.d.
IGF-I SDS	0	16	−2.0	0.9	6	−1.0	0.6	22	−1.8	0.9	12	−1.6	0.6	12	−1.5	0.9	24	−1.6	0.7
	1	16	−0.8	1.1	6	0.1	0.6	22	−0.6	1.1	12	0.5	1.1	12	0.4	1.0	24	0.5	1.0
	2	16	−1.0	1.0	6	0.3	1.2	22	−0.7	1.2	12	0.8	1.3	12	0.6	1.1	24	0.7	1.2
	3	16	−1.3	0.9	6	0.2	1.1	22	−0.9	1.1	12	1.1	1.6	12	0.5	1.7	24	0.8	1.7
	4	16	−1.3	1.0	6	0.5	1.3	22	−0.8	1.3	12	0.7	1.4	12	0.6	0.9	24	0.6	1.2
Change in IGF-I SDS from baseline	1	16	1.2	0.9	6	1.1	0.7	22	1.2	0.9	12	2.1	0.8	12	1.9	0.5	24	2.0	0.7
	2	16	1.0	0.9	6	1.3	1.1	22	1.1	1.0	12	2.4	0.8	12	2.1	0.8	24	2.2	0.8
	3	16	0.8	1.0	6	1.2	1.1	22	0.9	1.0	12	2.7	1.2	12	2.0	1.3	24	2.3	1.2
	4	16	0.8	0.8	6	1.5	1.0	22	1.0	0.9	12	2.3	1.0	12	2.1	0.7	24	2.2	0.8

*n*, number of patients with available data.

GH, growth hormone; IGF-I, insulin-like growth factor I; rhGH, recombinant human growth hormone; SDS, standard deviation score.

### Safety

**Figure 4:**
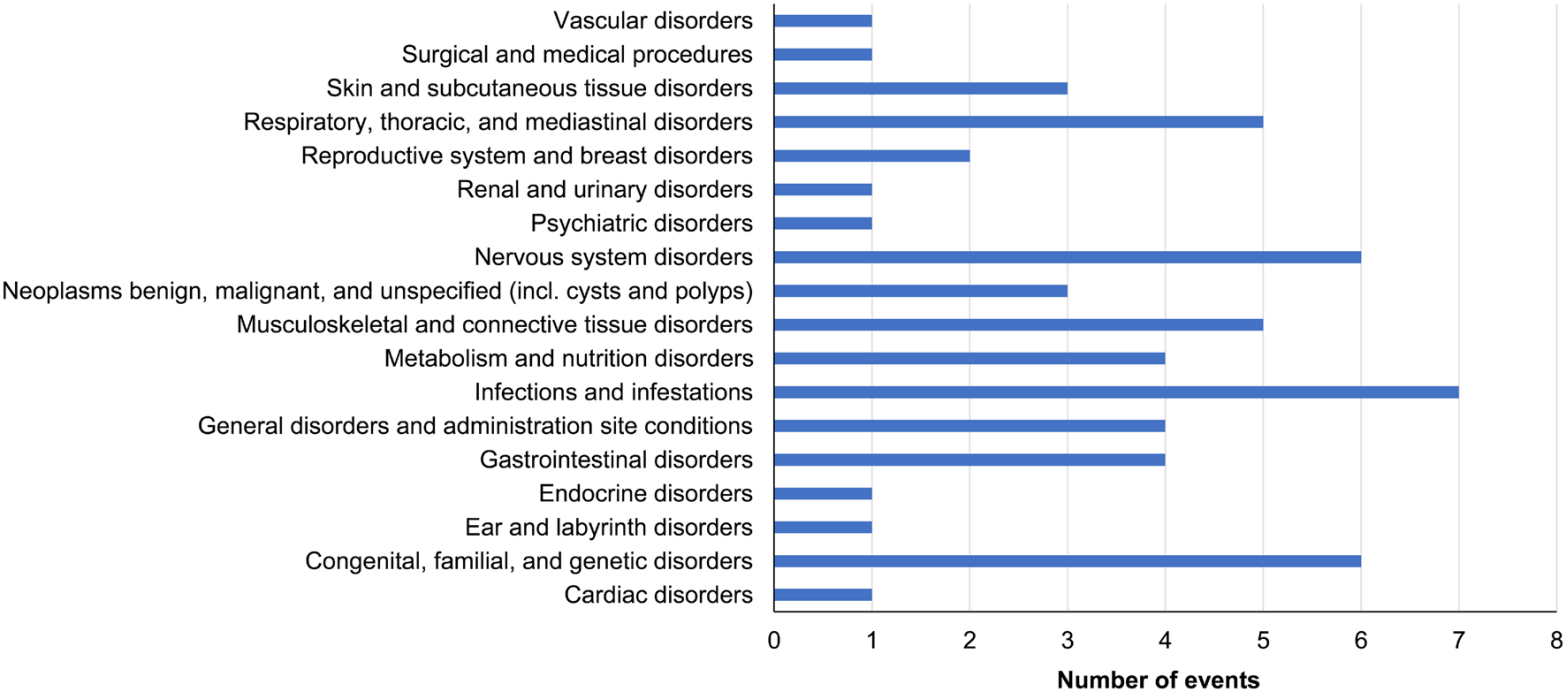
Safety events reported by MedDRA System Organ Class (safety analysis set, *n*  = 109). Data shown are the total number of events reported in each MedDRA System Organ Class, including non-SADRs, SADRs, and SAEs. MedDRA, Medical Dictionary for Regulatory Activities; SADR, serious adverse drug reaction; SAE, serious adverse event.

Overall, 38 patients (34.9%) from the safety analysis set reported an ADR or SAE. The total number of events is shown in Fig. 4 by Medical Dictionary for Regulatory Activities System Organ Class. The most frequently reported events included headache (five events reported in five patients) and arthralgia (three events reported in three patients). There was one SAE of atrial fibrillation reported in a 14‑year-old male patient who was *PTPN11* negative and *RIT1* positive, which has previously been described (25). Neoplasms were reported in two patients. Posterior fossa tumor and metastases to the spine were reported in a 15-year-old male patient who was *PTPN11* positive; a narrative of this event has previously been published (31). The second event was a brain neoplasm (dysembryoplastic neuroepithelial tumor grade 1) reported in a 9-year-old male patient who was *PTPN11* positive. The tumor diagnosis occurred 14 months after the patient commenced GHT. The patient discontinued GHT, was hospitalized, and underwent surgery to remove the mass from the right temporal lobe. It was judged as ‘unknown’ if the event was related to GHT, and the patient was reported to be recovering at the end of the follow-up period.

## Discussion

This analysis assessed the impact of *PTPN11* mutation status on long-term effectiveness outcomes in NS patients treated with rhGH. The effectiveness of GHT in treating short stature due to NS has been previously demonstrated (8, 29, 30, 31, 32, 33, 34). The impact of *PTPN11* mutation status on long-term GHT response has previously been discussed, but additional studies are needed to further clarify the role of the different RAS/MAPK pathway aberrations in growth and in GH responsiveness. Earlier studies reported that NS patients with a mutation in *PTPN11*display mild GH insensitivity, leading to reduced efficacy of short-term GHT (14, 35, 36). However, as NS patients with *PTPN11* mutations typically present with more pronounced short stature, it has been reported that although *PTPN11*-positive patients often reach a lower final height, the height gain following GHT is similar to that observed in *PTPN11-*negative patients (8).

Our effectiveness analysis included patients with clinically diagnosed NS, confirmed *PTPN11* mutation status, and who were GHT-naïve and pre-pubertal at baseline. Patient baseline characteristics were generally similar among *PTPN11*-positive and *PTPN11*-negative patients; the mean age at GHT start was the same for both groups and HSDS at baseline was also similar (−3.02 vs −3.09). In this analysis, we observed no significant differences in 4-year growth outcomes between *PTPN11*-positive and *PTPN11-*negative patients, in terms of HSDS and change in HSDS from the start of GHT.

The biological basis of growth failure in NS is not completely understood. There is some evidence that growth defects are related to partial GH insensitivity at a post-receptor level; using a mouse model of NS, it was demonstrated that NS-causing mutations in *PTPN11* inhibited IGF-I release via GH-induced extracellular signal-regulated kinase activation (37). Importantly, inhibition of this activation resulted in an increase of IGF-I levels *in vitro* and *in vivo* and was associated with significant growth improvements in NS mice (37). In patients, there have been no consistent changes reported in the GH–IGF-I axis in NS children. Various mechanisms have been hypothesized including GHD (12, 38), neurosecretory dysfunction (39, 40), and mild GH resistance (14). IGF-I levels have frequently been found to be low in NS patients, with levels often lower among *PTPN11*-positive patients compared with *PTPN11*-negative patients (14, 35, 36). It has also been reported that following 1 year of GHT, the increase in IGF-I was significantly lower in *PTPN11*-positive patients compared with* PTPN11*-negative patients (35), and a positive correlation was seen between growth velocity after the first year of GHT and the increase in IGF-I level (35).

Despite the apparent mild GH resistance suggested from short-term data, in which *PTPN11-*positive patients responded less efficiently to GHT than *PTPN11*-negative patients (14, 35, 36), longer-term data comparing adult height following GHT among* PTPN11*-positive and *PTPN11*-negative patients revealed no difference in final outcome between *PTPN11* mutation groups (31). In an IGF-I generation test study, 12 pre-pubertal NS children with mutations in *PTPN11* did display a blunted increase in IGF-I compared with 12 children with idiopathic short stature; however, the majority of the NS children did not display classic biochemical GH insensitivity (41). Evidence regarding GH insensitivity in NS patients who are *PTPN11* negative is particularly limited (16). Further studies are required to better understand growth failure in NS patients and the functional relationship between the various NS genotypes, GH sensitivity, and IGF-I signaling. In our analysis, we observed a greater increase in IGF-I SDS among patients who received the higher rhGH dose of 0.066 mg/kg/day in the GHLIQUID-4020 trial. Previous studies have demonstrated a positive correlation between rhGH growth response in patients with NS and an increase in IGF-I levels (23, 35); together, this suggests that higher rhGH doses may be required to overcome GH insensitivity and optimize growth outcomes in *PTPN11*-positive patients. However, patient adherence to rhGH and other factors that may affect IGF-I levels, for example, individual GH sensitivity, long-term fasting, and nutrition, were not assessed in this analysis, and therefore, this topic may require further investigation.

Increased GH resistance among *PTPN11*-positive NS patients is consistent with the finding that protein tyrosine phosphatase SHP-2 (encoded by *PTPN11*) has a role in GH signaling (37). *PTPN11* mutations can cause different degrees of increased tyrosine phosphatase activity in the SHP-2 protein (42). It was not possible to investigate the effect of each *PTPN11* mutation type on GHT response in the current analysis as this information was not collected, but this is an important topic for future research.

Previous studies investigating the effect of *PTPN11* mutation status on response to GHT have reported varied findings. In a retrospective analysis of 14 patients with NS (50% *PTPN11* positive) in Brazil monitored over 3 years of GHT, it was shown that GHT improved growth velocity for both groups, with greater gains in HSDS among *PTPN11-*negative patients. However, it should be noted that only a small number of patients reached the third year (*n*  = 4 in both groups) (35). Consistent with these findings, a French prospective study of 35 patients with NS (57% *PTPN11* positive) reported increased HSDS catch-up after 2 years of GHT among *PTPN11-*negative patients (36). A study of 15 pre-pubertal children with NS in Korea (60% *PTPN11* positive) reported that after 3 years of GHT, the improvement in HSDS was significantly greater among *PTPN11*-negative patients compared with patients with a *PTPN11*mutation (43). In another retrospective study that included 23 patients with NS (30% *PTPN11* positive) in Korea, patients without mutations in *PTPN11*, *RAF1*, *SOS1*, *KRAS*, or *BRAF*experienced greater improvements in HSDS, IGF-I SDS, and growth velocity compared with patients with a *PTPN11* mutation (19). However, a multicenter study of 29 NS patients (81% *PTPN11*positive among the 27 patients tested) across sites in the Netherlands and Belgium reported no difference in mean gain in HSDS between *PTPN11*-positive and *PTPN11*-negative patients over a median treatment duration of 6.4 years (range: 3.0–10.3 years) (8). Another retrospective study in Brazil that included 42 NS patients (83% *PTPN11* positive) reported a greater improvement in HSDS among *PTPN11*-positive patients after the first year of GHT, compared with *PTPN11*-negative patients (20).

In our analysis, BMI SDS was significantly lower among *PTPN11-*positive patients compared with *PTPN11-*negative patients, both at baseline and after 4 years of GHT. In line with this finding, a French national database study (*n*  = 420 NS patients) observed that patients with a *PTPN11* mutation were significantly shorter and thinner at birth compared with patients with other mutations (9), supporting the hypothesis that NS-associated mutations may impact the regulation of energy metabolism. A recent study investigated the lipid and glucose profile of pre-pubertal children (*n*  = 112) and adults (*n*  = 73) with NS and NS-related disorders, according to *PTPN11*mutation status. The study found that although the children had a lean phenotype, there was an increased frequency of low high-density lipoprotein cholesterol levels among *PTPN11-*positive patients (63% vs 59% in *PTPN11*-negative patients and 16% in control; *P* < 0.001) and impaired glucose metabolism (44). It is possible that the metabolic effects of a mutation in *PTPN11*could also impact patient IGF-I levels and response to GHT.

The safety data in our analysis are consistent with previous safety reports from rhGH-treated NS patients (23, 29, 32, 45). The most commonly reported events in our analysis were headache and arthralgia, which are well-known side effects of GHT in children. In addition, it has been suggested that migraines may be more prevalent in NS patients compared with the general population (46). There was one incident of atrial fibrillation in a patient with hypertrophic cardiomyopathy, although this was judged as unlikely related to GHT by the investigator and the patient recovered completely. There were two instances of brain neoplasms reported; however, in both cases, it was unknown whether the neoplasm was related to GHT and the history of headaches in one of the patients may suggest an underlying condition.

Up to 90% of patients with NS are reported to have concomitant cardiovascular abnormalities (47). In the current analysis, only 55% of patients had ≥1 cardiovascular comorbidity recorded at baseline. Among the *PTPN11*-positive patients, almost half did not have a concomitant cardiovascular diagnosis at the start of GHT. It is possible that these patients may have had minor cardiac abnormalities that had resolved over time, or that had been successfully treated prior to enrollment, and are, therefore, not captured in the analysis. In addition, patients with severe cardiac abnormalities were excluded from the GHLIQUID-4020 trial, which may help to explain the lower-than-expected prevalence of cardiac diagnoses in the analysis population (22).

The strengths of this analysis include the assessment of long-term outcome data over 4 years of GHT and that a relatively large number of patients were included, in comparison with previous studies of NS patients. The current analysis also included data collected from two geographic regions and from patients treated in a real-world setting, which may explain why some data were not available (e.g. provocative GH test results and limited details regarding some safety events). However, this analysis had some limitations that should be mentioned. First, potential confounding factors affecting growth outcomes, such as adherence to GHT and nutritional intake, were not taken into consideration. It is also possible that some of the *PTPN11*-negative patients included in our analysis may have been misdiagnosed with NS, as the diagnosis of NS may have been based only on clinical features if no other genetic mutations were identified. In addition, among patients with a confirmed *PTPN11* mutation, further details regarding the mutation variant were not collected. An independent evaluation of pathogenicity or differentiation between NS-associated and LEOPARD syndrome-associated variants was therefore not possible. Another potential limiting factor is the difference in cohort size between the *PTPN11-*positive and *PTPN11*-negative patients, which may have affected the statistical analysis. In addition, the *PTPN11*-negative group included patients with mutations in different genes with varying impacts on growth (e.g. the short stature phenotype of patients with *SOS1* mutation is usually milder than in patients with *RAF1* or *KRAS* mutations). A further limitation of this analysis is that the effect of GHT on patient final height could not be assessed as patients were not followed until final height was reached. Studies with a longer follow-up period are therefore needed in the future to investigate whether final height following GHT is impacted by *PTPN11*-mutation status.

Finally, there are some limitations found in all observational studies that may apply to the ANSWER program®; these include changes in diagnostic practices and the definition of eligibility for GHT during the study period (2002–2016) and a potential under-reporting of safety data.

## Conclusion

GHT over 4 years resulted in improved growth outcomes in GH treatment-naïve, pre-pubertal NS patients, irrespective of *PTPN11* mutation status. The long-term safety data are reassuring regarding the safety of GHT in this population, consistent with previous reports.

## Declaration of interest

The authors declare the following conflicts of interest in relation to this article: Alexander A L Jorge has received speaker fees from Novo Nordisk, Springer Healthcare, Pfizer, and Merck; independent research grant from BioMarin; and has received consulting fees from Novo Nordisk. Thomas Edouard has received research support from Novo Nordisk, Pfizer, and Sandoz (paid to INSERM unit for research use). Mohamad Maghnie has received grant support from Pfizer and Merck Serono and consultancy honoraria and speaker fees from Merck Serono, Novo Nordisk, Pfizer, Sandoz, Ascendis, and Biomarin. Alberto Pietropoli is an employee of Novo Nordisk Health Care AG. Nicky Kelepouris is an employee of Novo Nordisk Inc and holds stocks in Novo Nordisk and Pfizer. Alicia Romano is a consultant for Ascendis Pharma and a speaker and consultant for Novo Nordisk. Martin Zenker has received speaker and consulting fees from Novo Nordisk and funding from the German Ministry of Education and Research (BMBF), project title German Network of RASopathy Research (GeNeRARe; grant number: 01GM1902A). Reiko Horikawa served as an advisory board member for Novo Nordisk, OPKO-Pfizer, Ascendis, and Lumos Pharma and has received lecturer fees from Novo Nordisk, Pfizer, JCR, and Sandoz.

## Funding

The studies included in this analysis were sponsored by Novo Nordisk http://dx.doi.org/10.13039/501100004191 and are registered on ClinicalTrials.gov (NCT01009905 and NCT01927861). The sponsor was involved in study design, collection, analysis, and interpretation of data, and the decision to submit the article for publication. The sponsor was also given the opportunity to review the manuscript for medical and scientific accuracy as well as intellectual property considerations.

## Availability of data and material

Individual participant data will be shared in datasets in an anonymized format. Data may be shared with bona fide researchers submitting a research proposal requesting access to data. The access request proposal form and the access criteria can be found at www.novonordisk-trials.com. The data will be made available on a specialized SAS data platform.

## Ethics approval

All centers included in the ANSWER program® were approved by the local ethics committee or institutional review board, in accordance with country-specific rules. The study was conducted in accordance with the Declaration of Helsinki, Guideline for Good Pharmacoepidemiology Practices, and regulatory requirements. The GHLIQUID-4020 trial was reviewed and approved by local institutional review boards and was conducted in accordance with the Declaration of Helsinki, ICH Good Clinical Practice (GCP), and the Ministry of Health and Welfare Ordinance on GCP (1997). All patients (and/or their guardians where the patient was a minor) provided written informed consent to participate in the studies.

## Author contribution statement

All authors contributed to study conception and/or design, data acquisition, analysis, and/or interpretation; drafted the article or revised it critically for intellectual content; agreed to submit to the current journal; gave final approval of the version to be published; and agreed to be accountable for all aspects of the work.
